# Frequency detection of *BRAF* V600E mutation in a cohort of pediatric langerhans cell histiocytosis patients by next-generation sequencing

**DOI:** 10.1186/s13023-021-01912-3

**Published:** 2021-06-11

**Authors:** Shunqiao Feng, Lin Han, Mei Yue, Dixiao Zhong, Jing Cao, Yibing Guo, Yanling Sun, Hao Zhang, Zhenhua Cao, Xiaodai Cui, Rong Liu

**Affiliations:** 1 Department of Hematology, Children’s Hospital of Capital Institute of Pediatrics, Beijing, 100020 China; 2 Running Gene Inc, Beijing, China; 3 Department of Key Laboratory, Children’s Hospital of Capital Institute of Pediatrics, Beijing, 100020 China

**Keywords:** Langerhans cell histiocytosis, *BRAF* V600E mutation, Next-generation sequencing, Pediatrics, Biopsy tissue

## Abstract

**Background:**

Langerhans cell histiocytosis (LCH) is a rare neoplastic disease that occurs in both children and adults, and *BRAF* V600E is detected in up to 64% of the patients. Several studies have discussed the associations between *BRAF* V600E mutation and clinicopathological manifestations, but no clear conclusions have been drawn regarding the clinical significance of the mutation in pediatric patients.

**Results:**

We retrieved the clinical information for 148 pediatric LCH patients and investigated the *BRAF* V600E mutation using next-generation sequencing alone or with droplet digital PCR. The overall positive rate of *BRAF* V600E was 60/148 (41%). The type of sample (peripheral blood and formalin-fixed paraffin-embedded tissue) used for testing was significantly associated with the *BRAF* V600E mutation status (p-value = 0.000 and 0.000). The risk of recurrence declined in patients who received targeted therapy (p-value = 0.006; hazard ratio 0.164, 95%CI: 0.046 to 0.583). However, no correlation was found between the *BRAF* V600E status and gender, age, stage, specific organ affected, *TP53* mutation status, masses close to the lesion or recurrence.

**Conclusions:**

This is the largest pediatric LCH study conducted with a Chinese population to date. *BRAF* V600E in LCH may occur less in East Asian populations than in other ethnic groups, regardless of age. Biopsy tissue is a more sensitive sample for *BRAF* mutation screening because not all of circulating DNA is tumoral. Approaches with low limit of detection or high sensitivity are recommended for mutation screening to avoid type I and II errors.

**Supplementary Information:**

The online version contains supplementary material available at 10.1186/s13023-021-01912-3.

## Background

Langerhans cell histiocytosis (LCH) is inflammatory neoplasia of the myeloid precursor cells. The disease is characterized by the clonal proliferation of CDa1 + /CD207 + dendritic cells, whose features are similar to those of epidermal Langerhans cells. LCH is the most common histiocytic disorder but is a rare neoplastic disease. LCH presents in patients of all ages but most prevalent in children, with 3.5 years old being the medium age of diagnosis [1]. Generally, the incidence rates are 4 ~ 8 children per million and 1 ~ 2 adults per million each year, similar to those of pediatric Hodgkin’s lymphoma [1–4]. The highest incidence rate is observed among infants less than one year of age [1]. LCH lesions can develop in almost all systems but have a particular affinity for the skeleton (80%), skin (33%) and pituitary gland (25%) in children [1, 5]. The clinical manifestations depend on the specific organs involved and the extent of involvement, varying remarkably from spontaneously regressing lesions in isolated organs to fatal diseases in multiple systems [3]. The diversity of the symptoms contributes to the high misdiagnosis rates (16%), further leading to delays in appropriate treatment [1, 6]. The classification of LCH is based on the number of lesions present and organs affected. Approximately two-thirds of children present with single-system involvement, which has an excellent prognosis and a 5-year survival rate of virtually 100%. However, multisystem LCH, involving two or more organs, frequently has an unpredictable course. LCH involving risk organs at diagnosis is considered a high-risk disease, especially with organ dysfunctions [1, 7].

*BRAF* is considered one of the most common and well-known mutated kinases in human cancer, with driver mutations in several cancers, including melanoma, colorectal cancer, thyroid cancer and non-small cell lung cancer [8–11]. Its V600E mutation accounts for more than 90% of *BRAF*-activating mutations [2]. Studies have shown that *BRAF* V600E mutations are present in up to 64% of LCH samples [12]. Further research demonstrated that the *BRAF* mutation in LCH lesions significantly elevates phospho-extracellular signal-regulated kinase (ERK) expression, suggesting the activation of the mitogen-activated protein kinase (MAPK) pathway, which further suppresses cell migration and augments cell survival [13, 14]. However, the clinical significance of *BRAF* V600E in LCH is still contradictory. The low incidence and misdiagnosis of LCH limits studies in a large number of LCH patients. This article presents a retrospective single-center study on a cohort of pediatric LCH patients aimed at determining the frequency of mutations and clarifying the associations between the clinical features and *BRAF* V600E mutation in LCH.

## Results

### Clinical information

**Fig. 1:**
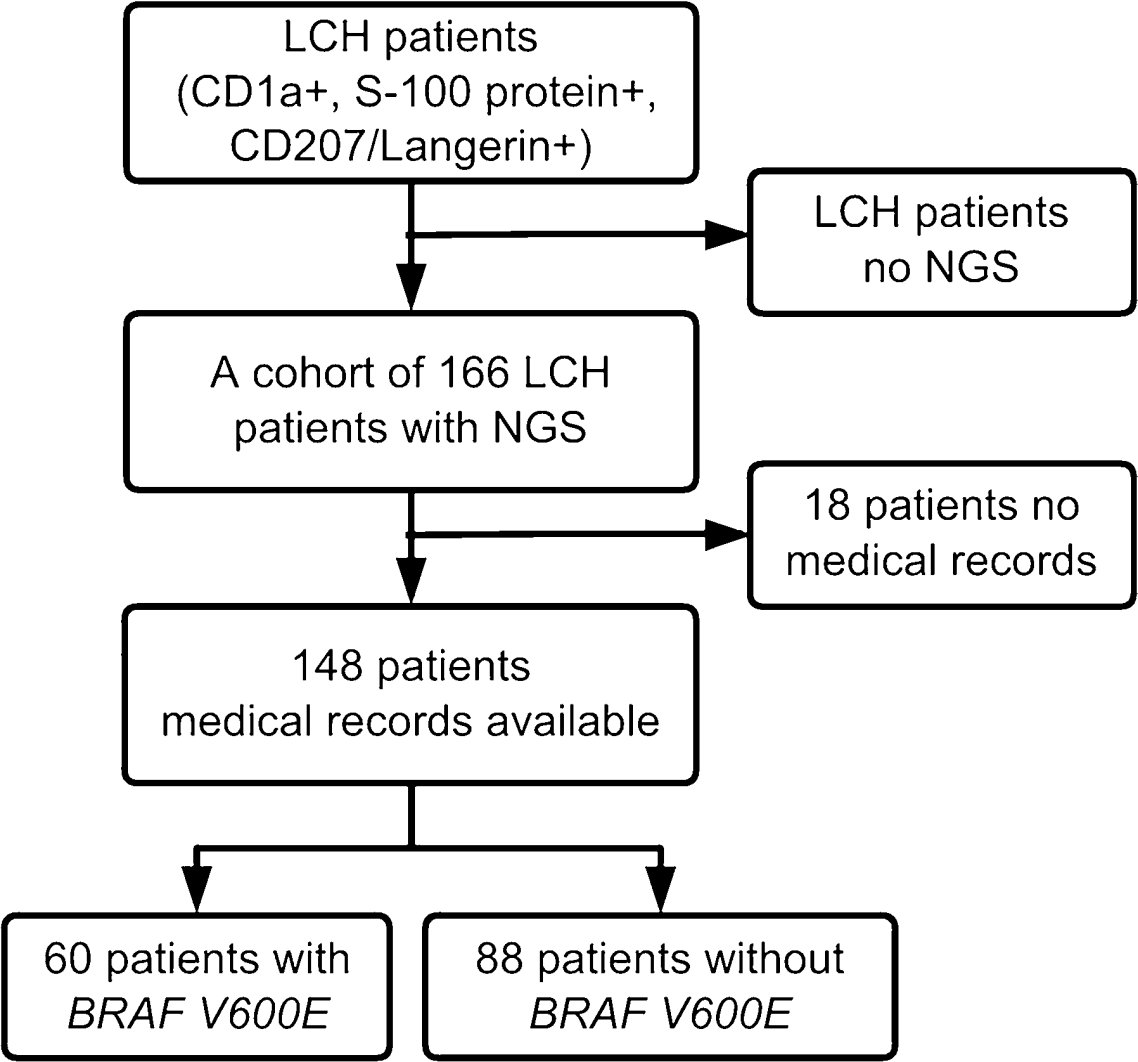
A flow chart of patient recruitment. A cohort of 166 LCH patients tested with next-generation sequencing (NGS) was recruited. Among them, the medical records of 148 LCH patients were available. Sixty of 148 patients showed *BRAF* V600E positivity in NGS. *BRAF* V600E mutation was identified in 60/148 (41%) pediatric LCH patients

**Fig. 2:**
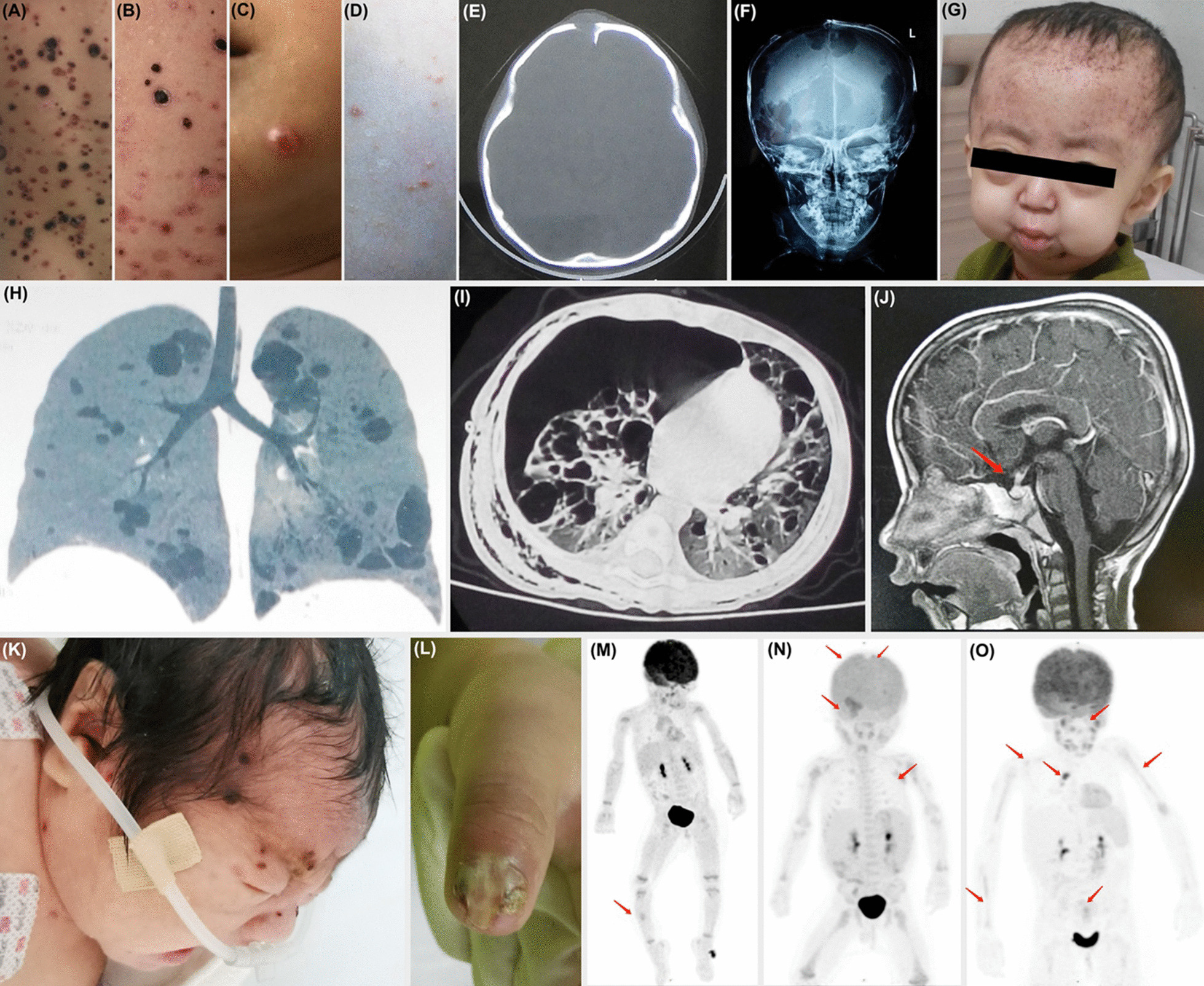
Spectrum of clinical presentations in Langerhans Cell Histiocytosis (LCH). **A**–**D** Different skin lesions. **E**, **F** Cranial bone lesions on single and multiple sites. **G** LCH patient with cranial and mandibular bone lesions and swollen eyes. **H**, **I** Lung lesions. **J** Pituitary lesions (arrow). **K** Skin lesions at birth. **L** Nail lesions. **M**–**O** PET-CT images showed a single bone lesion involving the right tibia; multiple bone lesions; multiple-system lesions involving the bones, lung and lymph nodes (arrow)

A total of 148 patients diagnosed with LCH were included in this research (Fig. 1). All were pediatric and of Chinese origin. The pertinent characteristics of patients are summarized in Table 1. The cohort included 88 boys and 60 girls, with a gender ratio of 1.47. The median and mean onset ages were 2 and 3.3 years old (range 0–16 years old), respectively. The onset age for more than half of the patients (57%) was equal to or less than two years old. Patients with the highest incidence were those younger than 1-year-old (29%). Seventy patients (47%) had single-system involvement, specifically 44 patients with single-site lesions (SS-S) and 26 patients with multiple-site lesions (SS-M). Seventy-eight patients (53%) had multiple-system involvement, specifically 57 patients without risk-organ involvement (MS-RO^−^) and 21 patients with risk-organ involvement (MS-RO^+^). Bone (79%) was the most common location of LCH involvement, followed by the skin (41%), lungs (26%), lymph nodes (14%), liver (14%), spleen (8%), pituitary gland (7%), bone marrow (4%) and thymus (3%). The imageology for the involvement of various organs is presented in Fig. 2. In addition, 71 patients (48%) had masses thought to be associated with LCH, including eosinophilic granuloma (10/71), lymphadenopathy (10/71) or extra-lymphatic masses (bone, 56/71; neck, 2/71; gingiva, 1/71; soft tissue in the nasal cavity, 1/71; medulla oblongata, 1/71; thymus, 1/71). According to the latest follow-up data, 41 of 136 LCH patients (30%) had relapsed. After treatments using various protocols (chemotherapy, targeted therapy, surgery or no treatment), LCH improved in 127 of 136 patients (93%). Two LCH patients, who only had skin lesions and recovered without receiving any treatments, were also included. Nearly all of the patients in the cohort are still alive, with a 1-year overall survival rate of 100% and 5-year of 99%. Only one patient had LCH concurrent with acute lymphoid leukemia and eventually died of peripheral T-cell lymphoma, although her LCH symptoms improved.

**Table 1:** Clinicopathological presentations of LCH patients

	Total	*BRAF* V600E	Without^1^	p-value, significance^2^
n (%)	148	60 (41%)	88	
*Gender, n (%)*				0.689, ns
Male	88 (59%)	34 (39%)	54	
Female	60 (41%)	26 (43%)	34	
*Onset age, n (y, %)*				0.575, ns
Median (years, range)	2 (0–16)	2 (0–11.8)	2 (0–16)	
< 1	43 (29%)	18	25	
1 ~ 2	41 (28%)	17	24	
3 ~ 5	39 (26%)	17	22	
> 5	25 (17%)	8	17	
*Stage, n (%)*				0.154, ns
SS-S	44 (30%)	17 (39%)	27	
SS-M	26 (18%)	9 (35%)	17	
MS-RO^−^	57 (39%)	21 (37%)	36	
MS-RO^+^	21 (14%)	13 (62%)	8	
*Organ involved*^*3*^*, n (%)*				
Bone	117 (79%)	46 (39%)	71	0.701, ns
Skin	61 (41%)	28 (46%)	33	0.346, ns
Lung	38 (26%)	13 (34%)	25	0.465, ns
Lymph node	21 (14%)	6 (29%)	15	0.368, ns
Liver	20 (14%)	8 (40%)	12	1.000, ns
Spleen	12 (8%)	6 (50%)	6	0.546, ns
Pituitary gland	11 (7%)	3 (27%)	8	0.527, ns
Bone marrow	6 (4%)	3 (50%)	3	0.683, ns
Thymus	5 (3%)	1 (20%)	4	0.648, ns
*Sample type*^*4*^*, n (%)*				
Peripheral blood	78 (53%)	18 (23%)	60	0.000, ***
FFPE tissue	84 (57%)	45 (54%)	39	0.000, ***
*Mutant TP53, n (%)*				0.572, ns
+	72 (49%)	27 (38%)	45	
−	76 (51%)	33 (43%)	43	
*Masses close to the lesion, n (%)*				1.000, ns
+	71 (48%)	29 (41%)	42	
−	77 (52%)	31 (40%)	46	
*Recurrence, n (%)(out of 136)*				0.407, ns
+	41 (30%)	11	30	
−	95 (70%)	45	50	
*Clinical outcome, n (%)(out of 136)*				–
Improved	127 (93%)	49	78	
Not	9 (7%)	6	3	

^1^Includes one patient with next-generation sequencing failure

^2^ns, not significant; ***, p < 0.001, statistically significant (also marked in bold)

^3^Correlations between the involvement of each organ and mutation status were analyzed individually

^4^Both peripheral blood and FFPE tissue were used for 14 patients

### Mutation analysis

The positive rates in next-generation sequencing (NGS) with LCH-panel were 28% (22/78) and 74% (62/84) in peripheral blood and formalin-fixed paraffin-embedded (FFPE) tissue samples, respectively. A group of genes was detected in our LCH cohort, including *ARAF*, *BRAF*, *MAP2K1*, *KRAS*, *KIT*, *FGFR3*, and *ALK* (unpublished data will be described in the next paper). *BRAF* V600E mutation was detected in 60 of 148 (41%) patients, based on the results of both LCH-panel NGS and droplet digital PCR (ddPCR) (Table 1). The NGS detection rate was 14% (11/78) in peripheral blood, with a V600E positive rate of 23% (18/78). In FFPE tissue samples, the NGS detection rate was 52% (44/84), with a V600E positive rate of 54% (45/84). Of the 14 patients in whom both types of samples were used for LCH-panel sequencing, the positive rates were 14% (2/14) for peripheral blood and 50% (7/14) for FFPE tissue, showing a low level of concordance between the two types of samples. Mutations in FFPE tissues were detected in bone and adjacent soft tissue (27/58), skin (15/22), lymph nodes (1/2), soft tissue on the neck (1/2), bone marrow (1/1) and soft tissue in the nasal cavity (1/1). No *BRAF* V600E mutation was detected in the gingiva (0/1) and thymus (0/1). NGS also revealed that 72 patients (49%) carried a *TP53* mutation (c.98G > C, p.P33R).

### Correlation of BRAF V600E mutation status and clinicopathologic characteristics

**Fig. 3:**
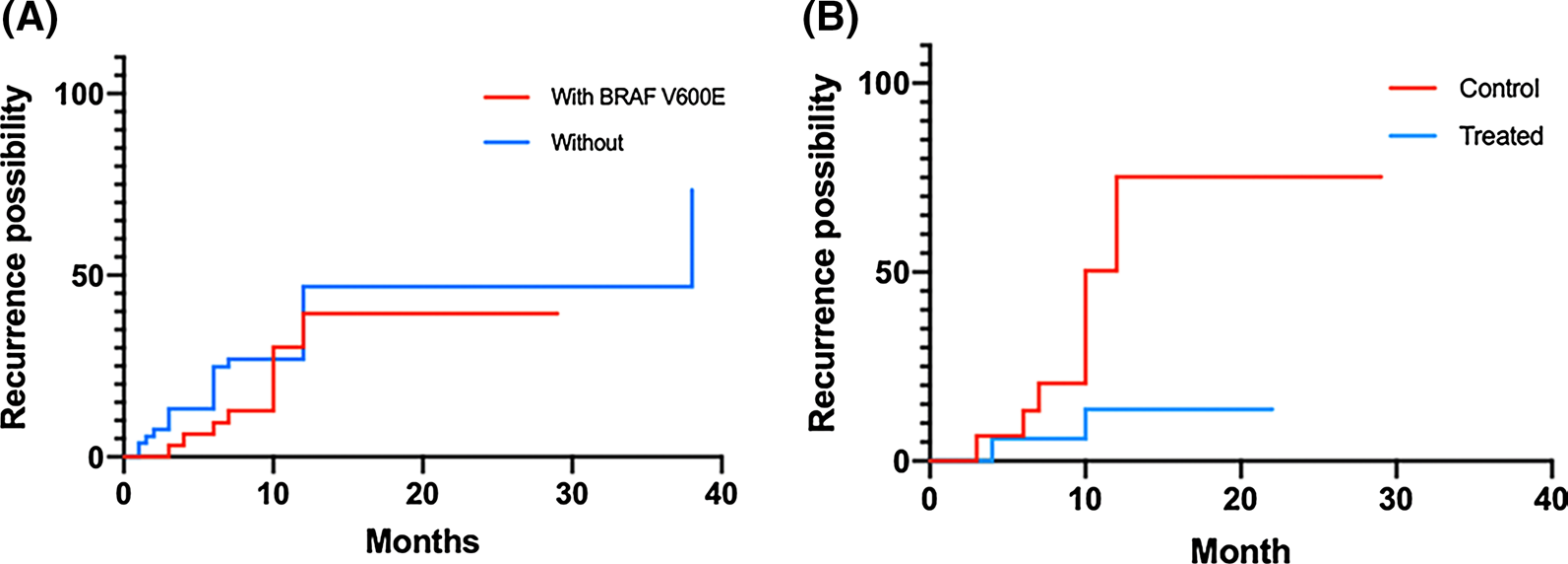
Recurrence possibility for *BRAF* V600E status and targeted therapy. **A** The relationship between recurrence and the mutation statue of *BRAF* V600E (Log-rank (Mantel-Cox) test, p-value = 0.407 > 0.05, not statistically significant). **B** Patients who received targeted therapy had a lower risk of recurrence (Log-rank (Mantel-Cox) test, p-value = 0.006 < 0.05, statistically significant; hazard ratio 0.164, 95%CI: 0.046 to 0.583)

The type of sample sent for sequencing was significantly correlated to *BRAF* V600E (p-value = 0.000 (2.66E−04) in peripheral blood and p-value = 0.000 (4.15E−04) in FFPE tissue). V600E mutations were detected in peripheral blood at a lower percentage (odds ratio 0.274, 95% confidence interval (CI) 0.128–0.567) than in FFPE tissues (odds ratio 3.734, 95% CI 1.741–8.341). The sensitivity was 61% (11/18) and 98% (44/45) for peripheral blood and FFPE tissue, respectively. However, there was no statistically significant correlation between the *BRAF* V600E status and gender (p-value = 0.689 > 0.05), onset age (p-value = 0.575 > 0.05), stage (p-value = 0.154 > 0.05), specific organ affected (all p-values > 0.05), *TP53* mutation status (p-value = 0.572 > 0.05), the presence of masses close to the lesion (p-value = 1.000 > 0.05) or recurrence (p-value = 0.407 > 0.05) (Fig. 3A) (Table 1). In our cohort, 52% (31/60) of patients harboring *BRAF* V600E were given targeted therapy, including but not limited to dabrafenib (30/31). The recurrence rate was 7% (2/29) in patients given targeted therapy and 33% (9/27) in patients that were not. Targeted therapy was statistically significantly associated with a decreased risk of recurrence in LCH patients (p-value = 0.006 < 0.05; hazard ratio 0.164, 95%CI: 0.046 to 0.583) (Fig. 3B).

## Discussion

### BRAF V600E frequency in LCH

Since *BRAF* V600E mutation in tissues was detected in almost all studies evaluated, only the frequencies of detection in tissues were compared and discussed here. In all reported studies, the frequency ranged from 0 to 64%, with an overall frequency of 47% (Table 2). In the current study, *BRAF* V600E was detected in 54% of LCH tissues specimens, consistent with that in the references. For patients < 18 years old, regardless of whether ethnicity was taken into account, *BRAF* V600E mutation was more frequent in them than in adults. The frequency in our pediatric cohort (54%) was consistent with the overall pediatric frequency (53%). In addition, when we analyzed frequency according to geographical factors, patients from East Asia showed a lower frequency than other ethnic groups, regardless of age. We therefore hypothesize that the frequency of *BRAF* V600E mutation varies across ethnic groups. Previous studies have also shown that ethnic background appears to influence the risk of LCH development [15]. However, the reasons for these differences are uncertain. The recruitment criteria, sample size, techniques used or the definition of various factors may all contribute to these discrepancies. For example, only samples with a variant allele frequency (VAF) greater than 4% were considered mutant in Badalian-Very’s study [16], but only samples with a VAF less than 0.1% were considered wild-type in our study. The definitions of mutation are inconsistent between studies. If we apply 4% as the threshold for VAF to analyze NGS data from our tissues, 49% (41/84) of patients are classified as *BRAF* V600E-positive (52% when the threshold for VAF is 0.1%), which is much less frequent than in Badalian-Very’s study (57%). In further research, setting identical recruitment criteria, increasing the sample size, unifying a standard testing approach and clarifying the definition of important factors can be considered to figure out the discrepancies.

**Table 2:** Summary of published cohorts of LCH patients bearing *BRAF* V600E mutations in tissue

References	Race	*BRAF* V600E, (n%)	*BRAF* V600E in patients < 18 years, (n%)	Median age, range (year)	Gender ratio (M:F, ratio)	Sample type(s)—Method(s)	Features assessed with *BRAF* V600E	Feature(s) with significance
Present cohort	Chinese	45/84 (54%)	45/84 (54%)	2 years, 0–16 years	88:60, 1.47	FFPE tissue—NGS, 44/84 (52%); NGS and ddPCR, 45/84 (54%) Circulating DNA—NGS, 11/78 (14%); NGS and ddPCR, 18/78 (23%)	Onset age, gender, stage, involved organ, sample type, masses close to the lesion, *TP53* mutation, recurrence	Sample type
Tong [21]	Chinese	0/18 (0%)	0	28.5 years, 18–78 years	14:4, 3.50	FFPE tissue—Direct sequencing	NA	NA
Zeng [22]	Chinese	36/97 (37%)	26/65 (40%)	10 years, 1–63 years	63:34, 1.85	FFPE or PE tissue—Sanger sequencing, 31/97 (32%) FFPE or PE tissue—IHC, 36/97 (37%)	Disease-free survival, PDL1, FOXP3 + Tregs, GATA3 + /T-bet + ratio	Disease-free survival, PDL1, FOXP3 + Tregs
Zeng [13]	Chinese	Same as group (22)					Age, gender, anatomic sites, stage, survival, recurrence	Age, recurrence
Liu [43]	Chinese	27/36 (75%)	19/23 (83%)	6 years, 1–56 years	23:13, 1.77	PE tissue—Sanger sequencing, 26/36 (72.2%) PE tissue—IHC, 25/31 (80.6%)	Age, sex, anatomic sites, stage, outcome	None
Sasaki [44]	Japanese	4/19 (21%)	4/16 (25%)	2 years, 0–71 years	9:10, 0.90	FFPE tissue—IHC and direct sequencing	NA	NA
Hayase [45]	Japanese	27/59 (46%)	24/50 (49%)	2.9 years, 0.3–17 years (children) 40.8 years, 19–67.1 years (adults)	34:25, 1.36	Fresh frozen and FFPE tissue—AS-qPCR and NGS	Sex, age at diagnosis, disease extent, response to first-line therapy, relapse, or CNS-related sequelae	MAP2K1 exon 2 in-frame deletion was related to the risk organ involvement
Kobayashi [23]	Japanese	9/23 (39%)	NA	42 years, 1–79 years	23:30, 0.77	Frozen or FFPE tissue—IHC Cell-free DNA—AS-qPCR and ddPCR, 6/33 (18%)	NA	NA
Go [46]	Korean	6/27 (22%)	NA	NA, 0–50 years	NA	FFPE tissue—Sanger sequencing and AS-qPCR	Age, gender, anatomic sites, stage, outcome, clinicopathological features	None
	East Asian population	154/363 (42%)	118/238 (50%)		254:176, 1.44			
Alayed [47]	American	8/50 (16%)	NA	36.5 years, 1–78 years	28:22, 1.27	FFPE tissue—Pyrosequencing	Age, gender, anatomic sites, stage, overall survival	Age
Badalian-Very [16]	American	35/61 (57%)	22/31 (71%)	12 years, 0.9–61 years	39:22, 1.77	FFPE tissue—Pyrosequencing and/or OncoMap mass spectrometric genotyping	Age, gender, anatomic sites, stage	Age
Ballester [32]	American	15/26 (58%)	15/26 (58%)	NA, 0.6–17 years	14:12, 1.17	FFPE tissue—AS-qPCR	NA	NA
Berres [12]	American	64/100 (64%)	64/100 (64%)	NA, 2–8 years	60:40, 1.50	FFPE tissue—AS-qPCR and/or langerin + cell—Sanger sequencing Circulating DNA—AS-qPCR, 17/77 (22%)	Age, gender, stage, CNS risk lesions, Diabetes insipidus	None
Brown [48]	American	18/40 (45%)	11/27 (41%)	11.5 years, 0–84 years	21:19, 1.11	FFPE tissue—AS-PCR and NGS	Age, gender, anatomic sites	None
Pina-Oviedo [49]	American	0/7 (0%)	0	54 years, 28–84 years	4:3, 1.33	FFPE tissue—Pyrosequencing, IHC and NGS	NA	NA
Roden [50]	American	19/54 (35%)	NA	27.6 ± 21.8 years (mean ± SD)	37:17, 2.18	FFPE tissue—IHC and validated by AS-PCR and Sanger sequencing	Cumulative tobacco exposure	Cumulative tobacco exposure in PLCH
	American population	159/338 (47%)	112/184 (61%)		203:135, 1.50			
Haroche [51]	French	11/29 (38%)	NA	NA, NA	NA	FFPE tissue—Pyrosequencing and IHC	NA	NA
Heritier [19]	French	173/315 (55%)	173/315 (55%)	3.2 years, 0–17.9 years	167:148, 1.13	FFPE tissue—Pyrosequencing or AS-qPCR or ddPCR or IHC	Age, gender, stage, involvement, follow-up years, 5-year relapse, death, permanent consequence, therapy	Median follow-up years, skin, risk organ, second-line therapy and/or rescue therapy requirement
Satoh [24]	French	9/16 (56%)	8/15 (53%)	7.595 years, 0–19 years	8:8, 1.00	Tissue—Pyrosequencing Whole blood—Pyrosequencing, 0/23 (0%)	Age of diagnosis, stage	None
Bubolz [18]	German	22/42 (52%)	NA	NA, (0.6–65 years)	22:20, 1.10	FFPE tissue—Pyrosequencing or BRAF 600/601 StripAssay and validated by Sanger sequencing	Age, gender, stage, anatomic sites, overall survival	CD1a
Sahm [52]	German and Austrian	34/89 (38%)	16/47 (34%)	22.5 years, 1–80 years	NA	FFPE tissue—IHC and direct sequencing	Age, proliferation rate (Ki-67), the activation of RAS pathway, strong expression of p53	None
Mehes [53]	Hungarian	8/15 (53%)	8/15 (53%)	4 years, 0–18 years	7:8, 0.875	FFPE tissue—AS-qPCR/direct sequencing and IHC	Original sites, time point, clinical and histologic features, overall survival	Overall survival
	European population	257/506 (51%)	205/392 (52%)		204:184, 1.11			
Overall		570/1207 (47%)	435/814 (53%)		661:495, 1.34			

AS, allele-specific; ddPCR, droplet digital PCR; FFPE, formalin-fixed paraffin-embedded; PE, paraffin-embedded; IHC, immunohistochemistry; NA, not available; NGS, next-generation sequencing

It has been reported that most (70%) LCH patients present with single-system involvement, ranging from 60 to 91.8% of patients, and several studies support this statement [1, 17–22]. However, in our cohort, only 48% of patients presented with single-system LCH. The uneven distribution of medical resources may explain the lower percentage in this study. Our hospital has advanced medical resources. Most of the patients travelled from less developed areas to seek medical advice in our hospital. Since most LCH patients with single-system involvement present mild symptoms and may not even require treatment, they are more likely to seek treatment in local hospitals. Thus, patients with severe disease have a higher probability of seeking medical treatment in our hospital, leading to a lower percentage of LCH patients with single-system involvement.

### Analysis of associations

Since previous studies showed contradictory findings on the correlations between mutation status and clinicopathological features, we aimed to clarify this relationship. In our cohort, the type of sample used for sequencing was significantly associated with the *BRAF* V600E mutation status. The frequency of mutations detected in FFPE tissues (54%) was much higher than that in peripheral blood samples (23%). In particular, for 14 patients whose peripheral blood and FFPE samples were both available, the positive rate of NGS for FFPE (50%, 7/14) was higher than that for blood (14%, 2/14). Similarly, the frequencies of mutations detected in peripheral blood samples were lower than that detected in tissues in several studies [12, 23, 24]. The concordance indicates that tissue is a relatively more reliable sample for identifying mutations. However, peripheral blood is not a useless sample for LCH. Blood from different periods can be used to detect circulating *BRAF* V600E to monitor disease progression over time [12], especially when lesions are recovered. Since the DNA isolated from peripheral blood is not exclusively tumoral and only a small amount of DNA can escape from the lesion into the circulation, it is reasonable to observe a relatively low positive rate in the blood.

Our data showed that the *BRAF* V600E mutation status was not associated with the risk of recurrence. Intriguingly, previous studies reported that *BRAF* V600E mutation was correlated with an increased risk of recurrence [12, 13]. In our cohort, LCH patients without *BRAF* V600E mutation appeared more likely to experience recurrence. We are inclined to attribute this result to targeted therapy, as we found a statisticall significant association between targeted therapy and a decreased risk of recurrence in patients with LCH. The lower risk of recurrence in patients carrying the V600E mutation may be due to the fact that they received timely and effective treatment. Since this is a retrospective study and patients received targeted therapy without professional guidance and medical supervision, we lack robust evidence to draw definitive conclusions regarding the effectiveness of targeted therapy on LCH. However, this study dose shed some light on the effectiveness of dabrafenib in the treatment of LCH. Dabrafenib and vemurafenib are specific to *BRAF* V600E/K mutation and were approved by the US Food and Drug Administration (FDA) for use in melanoma [25–27]. The FDA also approved vemurafenib for Erdheim-Chester Disease, another rare type of histiocytic neoplasm involving *BRAF* V600 mutation [28]. In recent studies, LCH patients carrying V600E also reportedly improved after vemurafenib administration alone or in combination with dabrafenib [28–30]. Thus, vemurafenib and dabrafenib have great potentials as targeted therapies for the treatment of LCH. A well-designed randomized clinical trial of single-agent dabrafenib to determine its effectiveness in LCH is planned and will be conducted in our institution soon.

The finding of no correlation between the *BRAF* V600E and *TP53* mutation status is inconsistent with the results of a previous study in which FFPE tissues were analyzed [31]. The lack of a correlation is probably due to the high number of patients without *BRAF* V600E in our cohort. Moreover, there were no statistically significant correlations between the mutation status and other parameters. It is possible that the lack of complete assessment of some lesions clearly described in the medical records may have affected the above statistical results. For example, masses considered to be associated with LCH were identified by clinical observation or radiography alone without a combined biopsy, so some masses close to bone lesions may have been classified as eosinophilic granuloma. Lymphadenectasis or the presence of liver lesions in some patients was diagnosed using abdominal ultrasound without biopsy. Hence, some symptoms may not be clearly classifiable or may not be caused by LCH.

### Approaches for BRAF V600E screening

The method used to evaluate mutation status can affect the results [32]. As shown in Table 2, immunohistochemistry (IHC, 10/21), Sanger/direct sequencing (10/21), allele-specific PCR (9/21) and pyrosequencing (7/21) are popular approaches for *BRAF* V600E screening but only a few studies applied NGS (4/21) or ddPCR (3/21). In our cohort, the limit of detection (LOD, n% mutant allele in a background of wild-type alleles) was 0.12% in ddPCR and 0.1% in LCH-panel NGS. However, the LOD for *BRAF* mutation can vary considerably across methods: IHC (5%), Sanger sequencing (6.6%), pyrosequencing (5%), NGS (1–2%), allele-specific qPCR (0.5%) and ddPCR (0.0005%) [32–35]. When using a method with a high LOD, false-negative results are likely to occur. Thus, approaches with low LOD should be prioritized to reduce type II errors in the future.

Compared to other approaches, ddPCR is a relatively inexpensive and sensitive technique but it does not distinguish between *BRAF* V600E and V600D, leading to false-positive errors in the detection. Our study used both NGS and ddPCR to confirm the mutation status and avoid the misuse of V600E/K inhibitors for V600D patients. In fact, two patients showed positive for V600E in ddPCR, but they actually carried V600D, which was detected by NGS. Thus, to prevent false-positive errors, we recommend performing *BRAF* mutation screening for LCH patients using a method with high specificity.

## Conclusions

This study detected the frequency of *BRAF* V600E mutation in FFPE tissue and peripheral blood samples from pediatric LCH patients of Chinese origin. The mutation is less frequent in children than in adults, and in East Asian populations than in other populations. We also found that tissue is a more reliable sample for genetic screening because only a small amount of DNA can escape from lesions into the circulation and not all of circulating DNA is tumoral. Blood sample is not favored for now, but it may have the potentials to contribute to the surveillance of disease progression in the future. The findings also suggest that dabrafenib is an effective drug for reducing the risk of relapse in LCH patients, though more rigorous studies are needed to verify its effectiveness. We recommend a low LOD or high sensitivity method for *BRAF* V600E mutation screening to avoid type I and II errors.

## Methods

### Patients and samples

**Fig. 4:**
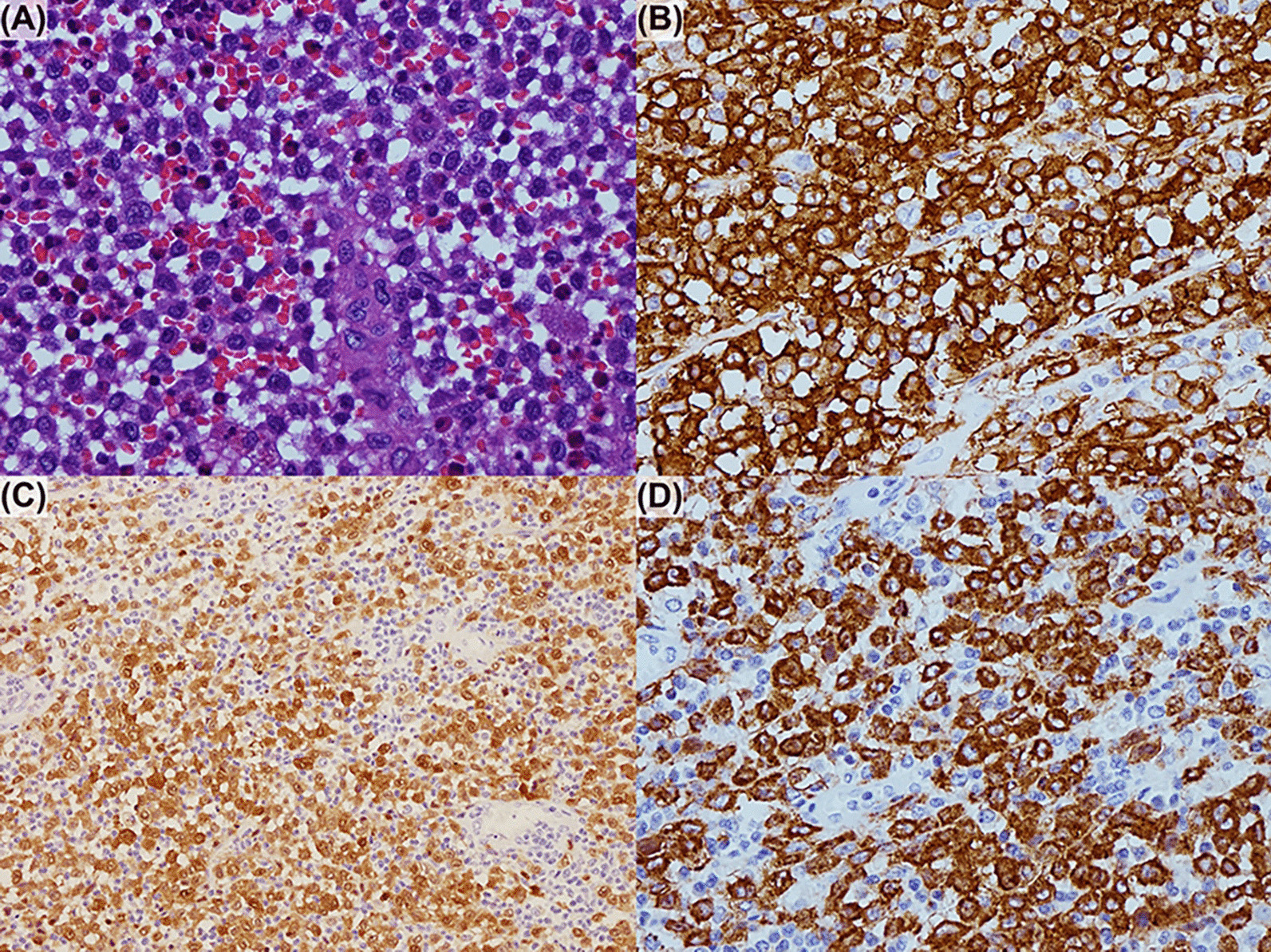
Histopathological staining of biopsies from LCH patients. LCH lesions with **A** hematoxylin and eosin staining (400 ×), **B** CD1a-positive immunostaining (400 ×), **C** S-100 protein-positive immunostaining (200 ×) and **D** CD207/Langerin-positive immunostaining (400 ×)

A cohort of LCH patients admitted to the Department of Hematology, Children’s Hospital of Capital Institute of Pediatrics in China from 2018 to 2019 was assembled in this study. Genetic information for a total of 166 patients was retrieved. The clinical and laboratory data for 92 patients were retrieved from their medical records, and data for 56 patients were collected through follow-up phone calls. Eighteen patients were excluded due to a lack of reliable clinical information. Therefore, the study focused on the 148 patients whose clinical and genetic data were available. The LCH diagnosis for all patients included was established through routine immunohistochemical examinations with positive CD1a, S-100 protein and CD207/langerin results (Fig. 4), according to the diagnostic criteria from the Histiocyte Society [36]. Based on the organ/system involvements, LCH is classified as affecting single system at single/multiple site(s) (SS-S/SS-M) or involving multiple systems without/with risk organ (MS-RO^−^/MS-RO^+^), which was defined as the bone marrow, liver or spleen [2]. LCH with risk-organ involvement is considered as high-risk, while other presentations are low-risk. This study was approved by the research ethics committee of the Children’s Hospital of Capital Institute of Pediatrics (Identifier: SHERLLM2020005). The guardians of the pediatric patients also signed written informed consent forms for the investigation and publication of articles.

### Next-generation sequencing

The NGS-LCH panel is commercially available by Running Gene Inc. (Beijing, China). A total of 29 genes involved in the RAF-ERK pathway and/or associated with LCH in previous studies were included in the panel (Additional file 1: Table S1 Gene list of LCH panel ).

Formalin-fixed paraffin-embedded (FFPE) tissue (n = 84) or/and peripheral blood (n = 78) samples from 148 LCH patients were collected, processed and sent for sequencing. Both FFPE tissue and peripheral blood samples were collected from 14 patients. Circulating DNA or DNA samples were isolated from the peripheral blood or FFPE tissues using the QIAamp Circulating Nucleic Acid Kit (#55114) or QIAamp FFPE DNA Tissue Kit (#56404, Qiagen, Hilden, Germany), respectively. The concentrations of extracted DNA were determined using NanoDrop One (#840-329700, Thermo Fisher Scientific, Waltham, MA) and the Qubit dsDNA HS Assay Kit (#Q32851, Invitrogen, Carlsbad, CA). Agarose gel electrophoresis or the DNA High Sensitivity Kit for Agilent 2100 Bioanalyzer (Agilent, Santa Clara, CA) was used for quality control. Qualified DNA samples were fragmented into 200 to 300 bp and then processed with the KAPA Hyper Prep Kit (Kapa Biosystems, Wilmington, MA) to build a DNA library. Customized probes were applied to perform a hybridization against the pooled libraries, and captured DNA fragments were enriched using PCR. The final products were sequenced on the Illumina HiSeq X10 platform (Illumina, San Diego, CA) with 150-bp paired-end reads. The raw average sequence depth was > 30,000 × for circulating DNA samples and > 500 × for tissue DNA samples.

Raw data were processed to fastq format by bcl2fastq v2.20 (Illumina, San Diego, CA). Software fastp v0.12.7 [37] was used for quality control and Burrows-Wheeler Alignment tool v0.7.16 [38] was used to map the pair-end reads to the human reference genome (GRCh37/hg19). The single nucleotide variants (SNVs) and insertions and deletions (INDELs) of targeted genes were called using GATK v3.7 [39] with MuTech2 [40]. Gene fusions were called using Genefuse version v0.5.0 [41]. Called variants were filtered in (reads > 5; depth > 500X) using snpEff v4.3 [42]. Filtered variants were annotated using an in-house annotation system based on public databases (1000 genomes project, ExAC, Exome Variant Server, dbSNP, COSMIC, My Cancer Genome, etc.). We identified several variants that were probably associated with LCH (unpublished data) but only focused on the *BRAF* V600E mutation here.

### Droplet digital PCR

The results of NGS for 69 patients were validated using ddPCR. Briefly, 2 × ddPCR Supermix for Probes (#1863028, Bio-Rad, Hercules, CA), 20 × assay PrimePCR™ ddPCR™ Mutation Assay Kit BRAF WT for p.V600E and BRAF p.V600E (#1863100, Bio-Rad, Hercules, CA), ddH_2_O and DNA templates were mixed well in a 20 μL reaction system, following the manufacturer’s instructions. Each reaction mix was loaded into a Droplet Generation 8 cartridge. Droplet Generation oil was loaded into the bottom row of the cartridge. After a 2-min running on the QX200 Droplet Generator (Bio-Rad, Hercules, CA), the droplets in the top row of the cartridge were collected and transferred into a 96-well plate. PCR amplification was performed with the T100 Thermal Cycler (Bio-Rad, Hercules, CA) following the instructions for the ddPCR Supermix for Probes. Afterward, the droplets were read and analyzed using the QX200 Droplet Reader (Bio-Rad, Hercules, CA).

### Statistical analysis

The frequency of *BRAF* mutations, along with the demographic information and assessed features for published cohorts of LCH patients bearing *BRAF* V600E mutations in tissue, were also summarized (Table 2). The frequency distributions of the clinicopathological data were collected and tabulated for patients with and without the *BRAF* V600E mutation (Table 1).

We hypothesized an association between each clinical characteristic and the mutation status (alpha cut-off of 0.05). The Chi-square test or Fisher’s exact test was used to determine the associations between clinical characteristics and the mutation status. Kaplan–Meier estimates and the Log-rank (Mantel-Cox) test were applied for recurrence and targeted therapy. P-values < 0.05 were considered statistically significant for all tests. Statistical analyses were performed with Microsoft Excel version 16.37 (Microsoft, Redmond, WA) and R (https://www.R-project.org/).

## Supplementary Information

**Additional file 1: Table S1**. Gene list of LCH panel.

## Data Availability

The datasets analyzed during the current study are not publicly available because the data is used by another undergoing study but are available from the corresponding author on reasonable request.

## Abbreviations

ddPCR: Droplet digital PCR
ERK: Extracellular signal-regulated kinase
FDA: Food and Drug Administration
FFPE: Formalin-fixed paraffin-embedded
INDELs: Insertions and deletions
LCH: Langerhans cell histiocytosis
MAPK: Mitogen-activated protein kinase
NGS: Next-generation sequencing
SNVs: Single nucleotide variants
SS-S: Single-system involvement at single site
SS-M: Single-system involvement at multiple sites
MS-OR^−^: Multiple-system without risk-organ involvement
MS-OR^+^: Multiple-system with risk-organ involvement
