# Supplementary material for: Frequency detection of BRAF V600E mutation in a cohort of pediatric langerhans cell histiocytosis patients by next-generation sequencing
Source: Orphanet J Rare Dis. 2021 Jun 11;16:272. doi: 10.1186/s13023-021-01912-3 (PMC8196454; doi:10.1186/s13023-021-01912-3)
Supplement: Supplementary file 1 — Additional file 1: Table S1. Gene list of LCH panel. [file 13023_2021_1912_MOESM1_ESM.docx]

**Supplementary File**

**Supplementary Table 1.** Gene list of Langerhans cell histiocytosis (LCH) panel

| AKT1 | ALK | ARAF | BRAF | CCND1 | CDKN2A |
| --- | --- | --- | --- | --- | --- |
| DDR2 | EGFR | ERBB2 | ERBB3 | FGFR1 | FGFR2 |
| HRAS | KIT | KRAS | MAP2K1 | MET | NOTCH1 |
| NTRK1 | PDGFRA | PIK3CA | PTEN | RAF1 | RET |
| ROS1 | SMO | TP53 | TSC1 |  |  |
